# IGF1 and PPARG polymorphisms are associated with reduced estimated glomerular filtration rate in a cohort of children and adolescents with type 1 diabetes

**DOI:** 10.1007/s00592-023-02128-6

**Published:** 2023-06-20

**Authors:** Chiara Zusi, Marco Rioda, Alice Maguolo, Federica Emiliani, Ilaria Unali, Silvia Costantini, Massimiliano Corradi, Giovanna Contreas, Anita Morandi, Claudio Maffeis

**Affiliations:** Department of Surgery, Dentistry, Gynecology and Pediatrics, Section of Pediatric Diabetes and Metabolism, University and Azienda Ospedaliera, Università di Verona, Piazzale A. Stefani, 1, 37126 Verona, Italy

**Keywords:** IGF1, PPARG, Diabetic nephropathy, T1D

## Abstract

**Introduction:**

Several genetic loci have been associated with diabetic nephropathy; however, the underlying genetic mechanisms are still poorly understood, with no robust candidate genes identified yet.

**Aim:**

We aimed to determine whether two polymorphisms, previously associated with renal decline, influence kidney impairment evaluating their association with markers of renal function in a pediatric population with type 1 diabetes (T1D).

**Material and methods:**

Renal function was evaluated by glomerular filtration rate (eGFR) and albumin-to-creatinine ratio (ACR) in a cohort of pediatric subjects with T1D (*n* = 278). Risk factors for diabetes complications (diabetes duration, blood pressure, HbA1c) were assessed. The *IGF1* rs35767 and *PPARG* rs1801282 SNPs were genotyped by TaqMan RT-PCR system. An additive genetic interaction was calculated. Association analysis between markers of renal function and both SNPs or their additive interaction were performed.

**Results:**

Both SNPs showed a significant association with eGFR: the A allele of rs35767 or the C allele of rs1801282 were associated to reduced eGFR compared to G alleles. Multivariate regression analysis adjusted for age, sex, *z*-BMI, T1D duration, blood pressure and Hba1c values showed that the additive genetic interaction was independently associated with lower eGFR (*β* = −3.59 [−6.52 to −0.66], *p* = 0.017). No associations were detected between SNPs, their additive interaction and ACR.

**Conclusions:**

These results provide new insight into the genetic predisposition to renal dysfunction, showing that two polymorphisms in *IGF1* and *PPARG* genes can lead to a reduction in renal filtration rate leading these patients to be exposed to a higher risk of early renal complications.

## Introduction

Diabetes Mellitus leads to several complications that can involve vessels, organs and peripheral nerves. Diabetic nephropathy (DN) is one of the most feared chronic complications of diabetes and the leading cause of chronic kidney disease (CKD) and end-stage renal disease (ESRD) [1–3]. Since DN is also an independent predictor of cardiovascular morbidity and mortality, the prevention and prediction of early signs of kidney dysfunction are a huge global challenge with important impact on health systems [4]. To date, no reliable predictors of renal survival are currently available for people with DN [5]. The decline of the estimated glomerular filtration rate (eGFR) and the presence of albuminuria are the typical hallmarks of kidney disease. However, these markers of renal function are not specific indicators of diabetes-related renal disease and may result in an inaccurate diagnosis [6].

Chronic hyperglycemia, duration of diabetes and hypertension are well-recognized predictors of DN, albeit the mechanisms involved in renal dysregulation are complex, and several molecular aspects are still unknown [6]. Indeed, the pathogenesis of DN is multifactorial and only in recent years mounting evidence demonstrated that various genetic and epigenetic factors contribute to its development and progression [7]. As regards the genetic basis of kidney functionality, recent studies supported the implication of insulin-like growth factors (IGF1 and IGF2) in several kidney diseases [8]. Particularly, a single nucleotide polymorphism (SNP, rs35767), located in the *IGF1* gene promoter region, resulted in the long-term chronological decline in renal function [9]. This SNP, which is a missense variation (-G1245A) directly affecting IGF1 expression. Specifically, carriers of the rs35767 T allele exhibit higher levels of circulating IGF1 [10, 11].

Another gene receiving increasing attention is the peroxisome proliferator-activated receptor gamma (*PPARG*) encoding a member of the ligand-activated nuclear hormone receptor superfamily. PPARγ agonists, indeed, exert protective effects against various kidney diseases including diabetic nephropathy [12]. The C/G transition of the rs1801282 within *PPARG* gene results in a proline to alanine exchange at amino acid position 12 (P12A) (rs1801282;Ex4-49C > G). This position is located in a PPARG domain responsible for ligand-independent activation of gene transcription [13]. The proline to alanine exchange may cause a conformational change in the PPARG protein and a reduced transcriptional activity of the rare alanine variant [14, 15]. Consistently, finding suggests that the Pro12Ala polymorphism (rs1801282) within *PPARG* gene can be associated to diabetic nephropathy, with Pro allele leading to increased levels of albuminuria and more elevated serum creatinine values in adults with type 2 diabetes [16].

Several studies used genetic scores with the aim of capturing the genetic variability associated with nephropathy [17–20], however, most of these researches focused on adults with type 2 diabetic nephropathy only [21, 22]. Although renal impairment occurs in both forms of diabetes (type 1 and type 2 diabetes), it is not known whether the genetic predisposition is conferred by the same genetic background. Moreover, it is currently uncertain whether the previously mentioned associations also occur in children and adolescents with T1D.

Therefore, the aim of this study was to test whether two genetic variants on *IGF1* and *PPARG* genes, known to influence the susceptibility to renal function decline, are associated with early index of renal impairment in children and adolescents with T1D.

## Methods

### Study population

Two hundred and seventy-eight children and adolescents with T1D (age: 14.5 ± 3.0 years, 146 boys) were consecutively recruited at the Regional Center for Pediatric Diabetes of the University Hospital, Verona (Italy) during a follow-up visit between January and December 2019. Inclusion criteria were diagnosis of T1D for at least one year and confirmed by positivity of at least two diabetes-associated autoantibodies (GADA, ZnT8A, IAA or IA–2A) and age between 6 and 18 years. Exclusion criteria were chronic diseases other than T1D, tubular or glomerular pathologies, chronic use of drugs other than insulin. Written informed consent to participate in the study was obtained from the parents/guardians of the children and adolescents. The study was approved by the Ethical Committee of the University Hospital of Verona.

### Clinical data collection

Patients underwent a physical examination to record anthropometric measurements (body weight and height) and blood pressure. Body mass index (BMI) was calculated using the formula: body weight (kg)/body height (m)^2^, values were then standardized (BMI *z*-score) calculating age and sex-specific BMI percentiles according to World Health Organization (WHO) child growth standards [23]. A physician measured the systolic blood pressure (SBP) and diastolic blood pressure (DBP) three times on the left arm with the subject sitting, using a digital sphygmomanometer and a cuff of appropriate size. The *z*-score of the average of the three blood pressure values, based on the European Hypertension Society references [24], was used for the analysis. All patients underwent a fasting blood test for measuring glycated hemoglobin (HbA1c) by high-performance liquid chromatography technique and standardized to the normal range established by the DCCT (4.0–6.0%, 20–42 mmol/mol).

### Estimation of kidney function

Estimated Glomerular Filtration Rate was calculated using Bedside-Schwartz’s equation for pediatric patients according to the following formula: 0.413 × body height (cm)/SCr (mg/dL) [25]. Serum creatinine (SCr) by enzymatic method was estimated. Evaluation of albuminuria excretion was determined in early morning urine samples as the albumin-to-creatinine ratio (ACR; units: mg albuminuria/mmol creatininuria).

### Genotyping and additive genetic interaction

Genomic DNA was extracted using a standard salting-out procedure from peripheral blood leukocytes of EDTA-anticoagulated blood. The genotypes of rs35767 and rs1801282 were determined by TaqMan allelic discrimination assay (Applied Biosystems, USA) according to manufacturer’s protocol using QuantStudio TM 5 Real-Time PCR (Applied Biosystems). Both genotyped SNPs were in Hardy–Weinberg equilibrium (HWE), and genotyping call rate was above 99% for each SNP. Minor allele frequency (MAF) for A-rs35767 was 18.5%, whereas G allele MAF of rs1801282 was 7.0%. An additive genetic interaction was created summing up the number of risk alleles in each patient: A allele of rs35767 within *IGF1* gene and C allele of rs1801282 within *PPARG* gene.

### Statistical methods

Data are presented as arithmetic mean with the relative standard deviation, medians, and interquartile range (IQR) or absolute and relative frequency. Normal distribution of variables was assessed by Kolmogorov–Smirnov test. Skewed variables were log-transformed, unless deviations from the Gaussian distribution could not be corrected by transformation. Differences among patients stratified by *IGF1* rs35767 genotypes and the additive genetic interaction were assessed by ANOVA or Kruskal–Wallis test, based on the Gaussian or skewed distribution of variables, while differences among *PPARG* rs1801282 genotypes were tested only by the nonparametric Kruskal–Wallis test because of the groups’ numerosity. Chi-square test was applied to detect differences among categorical variables. Renal function markers (eGFR and ACR) were further evaluated by linear regression analysis using the additive genetic interaction and covariates (age, gender, BMI *z*-score, T1D duration, SBP and DBP percentiles and Hba1c values) as independent variables. Covariates included in multivariate regression models were selected as potential confounding factors based on their biological plausibility. Significance level for all tests was set at *p* < 0.05. All analyses were performed using the IBM SPSS Statistics 26 statistical package (SPSS, Chicago, IL, USA).

## Results

A population of 278 pediatric patients with T1D (146 (53%) males and 132 (47%) females) with mean age of 14.5 ± 3.0 years was recruited. The main anthropometric and metabolic characteristics of the study sample are shown in Table 1.

**Table 1:** Main clinical and biochemical characteristics of children and adolescents with type 1 diabetes

	All (*n* = 278)
Age	14.52 ± 2.99
Years of diabetes	7.55 ± 3.58
Body height (cm)	161.1 ± 13.8
Body weight (kg)	56.45 ± 15.70
BMI	21.29 ± 3.65
BMI *z*-score	0.40 ± 0.90
SBP (mmHg)	107.55 ± 10.72
SBP percentile	36.0 [17.7–57.5]
DBP (mmHg)	68.25 ± 7.33
DBP percentile	60.6 [44.6–77.5]
HbA1c (%)	8.0 [7.4–8.5]
HbA1c (mmol/mol)	64 [57–69]
Serum Creatinine (mg/dL)	0.64 ± 0.15
eGFR (mL/min)	108.22 ± 20.17
ACR (mg/mmol) (*n* = 225)	0.37 [0.20–0.70]
*PPARG* rs1801282	
GG, *n* (%)	3(1.1)
CG, *n* (%)	33(11.9)
CC, *n* (%)	242(87.1)
*IGF1* rs35767	
GG, *n* (%)	191(68.7)
AG, *n* (%)	72(25.9)
AA, *n* (%)	15(5.4)
*PPARG* + *IGF1*	2.23 ± 0.70

Sample size, *n* = 278, otherwise indicated. Data are expressed as means ± SD, medians [IQR] or number and percentages

*BMI* body mass index, *SBP* systolic blood pressure, *DBP* diastolic blood pressure, *ACR* albumin-to-creatinine ratio

Tables 2 and 3 reported clinical and anthropometrics characteristics in study subjects according to *IGF1* (rs35767) and *PPARG* (rs1801282) genotypes, respectively. The SNP rs1801282 was differentially distributed between sexes: C allele was more represented in females than males. Both SNPs showed a significant association with eGFR in the analysis of the variances: subjects with recessive genotype for the minor allele (A) of rs35767 or subject carriers of the major allele (C) of rs1801282 have a reduced value of eGRF compared to the other genotypes (*p* = 0.033 and *p* = 0.046, respectively). No association was detected between any SNP and ACR. However, the A allele of rs35676 was associated with higher value of serum creatinine (*p* = 0.033) (Table 2 and 3).

**Table 2:** Clinical and anthropometrics characteristics in study subjects according to *IGF1* (rs35767) genotypes

	GG (*n* = 191)	AG (*n* = 72)	AA (*n* = 15)	*p* value
Age	14.40 ± 3.02	14.64 ± 3.06	15.50 ± 1.96	0.362
Males, *n* (%)	102 (53.4)	34 (47.2)	10 (66.7)	0.355
Years of diabetes	7.41 ± 3.67	7.60 ± 3.48	9.10 ± 2.73	0.210
Body height (cm)	160.4 ± 13.8	161.7 ± 14.1	167.6 ± 11.2	0.140
Body weight (kg)	55.82 ± 15.89	57.17 ± 15.89	61.00 ± 11.90	0.425
BMI	21.22 ± 3.65	21.40 ± 3.42	21.57 ± 3.09	0.888
BMI *z*-score	0.39 ± 0.87	0.47 ± 0.97	0.30 ± 0.83	0.947
SBP (mmHg)	107.6 ± 11.0	107.7 ± 10.4	106.8 ± 9.0	0.960
SBP percentile	36.4 [17.8–61.2]	32.8 [18.8–56.5]	33.0 [2.8–56.4]	0.608
DBP (mmHg)	68.0 ± 7.9	68.5 ± 5.7	70.5 ± 7.3	0.416
DBP percentile	61.8 [44.4–77.8]	58.6 [45.0–75.4]	68.0 [45.6–78.8]	0.728
HbA1c (%)	8.0 [7.4–8.5]	7.9 [7.3–8.5]	8.1 [7.5–8.4]	0.702
Serum creatinine (mg/dL)	0.63 ± 0.15	0.65 ± 0.15	0.73 ± 0.10	**0.033**
eGFR (mL/min)	109.8 ± 20.8	106.3 ± 18.9	96.7 ± 12.9	**0.033**
ACR (mg/mmol)	0.35 [0.20–0.67]	0.38 [0.20–0.80]	0.59 [0.24–1.22]	0.332

Data are expressed as means ± SD, medians [IQR]. Differences among groups were tested by ANOVA for normally distributed continuous variables, and the Kruskal–Wallis’s test for skewed variables

In bold statistically significant differencies are reported

*BMI* body mass index, *SBP* systolic blood pressure, *DBP* diastolic blood pressure, *ACR* albumin-to-creatinine ratio

**Table 3:** Clinical and anthropometrics characteristics in study subjects according to *PPARG* (rs1801282) genotypes

	CC (*n* = 242)	CG (*n* = 33)	GG (*n* = 3)	*p* value
Age	14.42 ± 3.00	15.26 ± 2.89	14.83 ± 2.33	0.309
Males, *n* (%)	121 (50.0)	25 (75.8)	0 (0)	0.004
Years of diabetes	7.49 ± 3.60	8.16 ± 3.39	6.02 ± 5.10	0.437
Body height (cm)	160.6 ± 13.7	165.0 ± 14.5	160.3 ± 5.5	0.130
Body weight (kg)	56.05 ± 15.78	58.67 ± 14.86	64.33 ± 20.60	0.380
BMI	21.27 ± 3.68	21.13 ± 3.12	24.66 ± 6.25	0.550
BMI *z*-score	0.41 ± 0.90	0.29 ± 0.88	0.95 ± 1.00	0.548
SBP (mmHg)	107.3 ± 10.3	108.8 ± 13.3	111.7 ± 18.9	0.693
SBP percentile	40.0 ± 26.4	36.5 ± 27.1	59.7 ± 47.5	0.489
DBP (mmHg)	68.4 ± 7.3	67.6 ± 7.5	67.7 ± 9.3	0.639
DBP percentile	59.8 ± 20.9	57.1 ± 21.0	58.1 ± 26.7	0.742
HbA1c (%)	8.0 ± 0.9	8.3 ± 0.9	8.3 ± 1.8	0.163
Serum creatinine (mg/dL)	0.64 ± 0.15	0.68 ± 0.16	0.51 ± 0.11	0.088
eGFR (mL/min)	108.4 ± 20.0	104.3 ± 20.0	133.8 ± 27.3	**0.047**
ACR (mg/mmol)	0.98 ± 1.99	0.90 ± 1.90	0.56 ± 1.56	0.512

Data are expressed as means ± SD. Differences among groups were tested by Kruskal–Wallis’s test

Bold value represents statisically significant result

*BMI* body mass index, *SBP* systolic blood pressure, *DBP* diastolic blood pressure, *ACR* albumin-to-creatinine ratio

The additive interaction of the two SNPs was significantly associated with eGFR values (*p* = 0.028): the higher the number of risk alleles, the lower the eGFR value. No association was detected between the genetic interaction and ACR. (Table 4).

**Table 4:** Median and IQR of markers of renal function according to the additive genetic interaction values

Risk allele number	0 (*n* = 1)	1 (*n* = 28)	2 (*n* = 168)	3 (*n* = 69)	4 (*n* = 12)	*p*-value
eGFR (mL/min)	155.8	102.7 [94.2–112.8]	109.8 [94.0–121.5]	104.5 [92.2–113.5]	95.4 [89.7–100.9]	**0.028**
ACR (mg/mmol)	0.42	0.29 [0.20–0.67]	0.36 [0.20–0.67]	0.38 [0.20–0.80]	0.72 [0.20–1.24]	0.554

Data are expressed as median [IQR]. Differences among groups were tested by Kruskal–Wallis’ test

Bold text indicates a statistically significant result

The multiple regression analysis showed similar results (Table 5). No association was highlighted between the additive genetic interaction and ACR (*p* = 0.952), whereas SNPs additive interaction resulted significantly associated with the variability of eGFR (*p* = 0.017, β-coefficient = −3.59 [−6.52 to −0.66]), independent of age, sex, T1D duration, BMI *z*-score, SBP, and DBP percentiles and HbA1c.

**Table 5:** Multivariate linear regression analysis in the total sample for markers of renal function (eGFR and ACR) using clinical and glycemic parameters and the additive genetic interaction (*PPARG* + *IGF1*) as independent variables

Dependent variable	Independent variables	β-coefficient [95% CIs]	*p*-value
eGFR (mL/min)	Age	−3.18 [−3.94 to −2.43]	** < 0.001**
	Sex	7.39 [3.23 to 11.6]	**0.001**
	T1D duration	−0.30 [−0.93 to 0.33]	0.348
	BMI *z*-score	−1.24 [−3.56 to 1.06]	0.290
	SBP percentile	−0.03 [−0.11 to 0.06]	0.521
	DBP percentile	0.06 [−0.05 to 0.17]	0.301
	HbA1c	19.43 [1.49 to 37.38]	**0.034**
	*PPARG* + *IGF1*	−3.59 [−6.52 to −0.66]	**0.017**
ACR (mg/mmol)	Age	−0.01 [−0.11 to 0.09]	0.803
	Sex	0.59 [0.48 to 1.14]	**0.033**
	T1D Duration	−0.02 [−0.10 to 0.07]	0.717
	BMI *z*-score	−0.16 [−0.47 to 0.15]	0.315
	SBP percentile	0.00 [−0.01 to 0.01]	0.978
	DBP percentile	0.00 [−0.01 to 0.02]	0.868
	HbA1c	0.61 [−1.76 to 2.98]	0.614
	*PPARG* + *IGF1*	0.01 [−0.38 to 0.40]	0.952

*R*^2^ = 0.310, *p* < 0.001

*R*^2^ = 0.028, *p* = 0.641

Statistical test performed on log-transformed HbA1c values

Bold values represent statistically significant results

*SBP* systolic blood pressure, *DBP* diastolic blood pressure

## Discussion

This is the first cross-sectional study aimed at examining the association between an additive genetic interaction of two SNPs with kidney function in a pediatric cohort with T1D, a group of subjects at high risk for developing microvascular complications, including DN.

The main and novel findings of our study were that the additive interaction, composed by two variants within *PPARG* and *IGF1* genes, was significantly associated with lower values of eGFR in a cohort of Italian children and adolescents with T1D. Other risk factors independently associated with reduced kidney function were male sex, older age, and higher HbA1c. Notably, the association between the genetic additive interaction and eGFR persisted also after adjustment for age, sex, and HbA1c.

This finding suggests that children with higher genetic risk have a lower baseline eGFR even before a diabetic related renal compromission could occur. The importance of glomerular filtration rate in predicting renal functionality has been previously demonstrated. Indeed, many studies concluded that filtration rate is a good predictor of major outcome: individuals with lower eGFR have an increased rate of mortality [26] and a higher value of EURODIAB PCS score, a score created to assess major vascular complications in patients with T1D [27].

Both eGFR and albuminuria exhibit familial aggregation, but eGFR appears to have higher heritability values and consequently a stronger genetic influence [28]. In concordance with this observation, we did not highlight any association between albuminuria and genes previously associated with chronic kidney disease [19, 20]. In addition, the natural history of diabetic nephropathy in T1D includes an asymptomatic phase in which albuminuria and blood pressure are normal, while the structural alterations that characterize diabetic glomerulopathy begin to develop [29]. Crucially, DN is not clinically detectable until significant kidney damage has developed, highlighting the need to identify early-stage biomarkers.

The *PPARG* gene represents an excellent bridge between the two types of diabetes: polymorphisms in PPARγ promoter regions contribute to the genetic predisposition to T1D and affect the severity of islet autoimmunity [30]. Additionally, PPARγ is associated with the development of insulin resistance and type 2 diabetes [31]. The major allele of rs1801282 SNP was previously associated with CKD and DN in subjects with T2D [32–35] and has already been reported to predict ESRD in patients with T1D [36].

The involvement of this molecule on renal impairment is confirmed by the fact that PPARγ agonists can slow or even prevent the progression of various kidney injuries including diabetic nephropathy via multiple mechanisms involving activation of PPARγ in the kidney and other tissue. PPARγ, the product of the *PPARG* gene, can be found in almost all type of cells in kidney both on vascular and parenchymal structures; this can explain the role that this gene plays in pathogenesis of DN, altering glomerular blood pressure due to a defected modulation in the production on renin in the juxtaglomerular cells and via reduction of the nitric oxide (NO) endothelial production [37, 38]. In addition, PPARγ inactivation can predispose to DN through the reduction of insulin sensitivity and the consequential worsen glycemic control, major risk factor for this complication [38, 39].

The IGF1 hormone, encoded by *IGF1* gene, plays an important role in modulating many physiological processes such as glucose and lipid metabolism, insulin sensitivity, and inflammatory response. The A-rs35767 risk allele was previously seen to be associated with a faster decrease of glomerular filtration rate and an increase in circulating level of IGF1 [9, 40], which in turn was found associated with a major risk of developing DN and chronic kidney diseases in general [41]. Alteration of glucose/insulin system, such as in subjects with diabetes, may increase both local synthesis and action of IGF1, leading to glomerular enlargement and progression of diabetic nephropathy [42].

Variants could contribute to identifying the subset of children and adolescents with diabetes and are at increased risk to progress to nephropathy and ESRD. Early identification will facilitate prevention and earlier intervention, ultimately delaying and reducing the severity of DN.

The limitations of our study are: (i) the assessment of smoking, which is a major risk factor for DN, was not available. However, the young age of the sample makes the potential impact of smoking negligible; (ii) ethnicity limited to Caucasian does not allow to export the results to children and adolescents with other ethnic backgrounds; (iii) the presence of individuals with renal hyperfiltration, that may influence these associations, cannot be totally excluded; (iv) a control group, composed of children and adolescents without type 1 diabetes, was not available; (v) the lack of a validation cohort suggests caution against the generalizability of our findings to other pediatric groups; *vi*. since the minor allele of rs1801282 has a very low frequency, despite being a missense variant, it might be useful to expand the analysis to other SNPs within the gene.

However, this study has some strengths: (i) the young age of the sample: children and adolescents with T1D have much lower diabetes-associated comorbidities than adults and, by implication, this offers the opportunity to explore genetic associations with DN with reduced influence of other potential confounding factors; (ii) our cohort has been recruited in a single center: cohort studies following patients from inclusion confer a high and uniform level of ascertainments.

## Conclusion

In conclusion, the present study shows that children and adolescents with T1D carrying the risk alleles A on *IGF1* (rs35767) and/or C on *PPARG* (rs181282) had a reduced eGFR, although within normal ranges. This result enhances the hypothesis that genetic variants on these genes can lead to a reduction in renal function leading these patients to be exposed to a higher risk of early renal complications. Gene-environmental synergy is more important than either single factor alone, and combining genetic and clinical information might be useful for risk stratification in clinical practice.

Further studies, especially with a longitudinal design, are needed to confirm this hypothesis and the implication of these genetic variants in the predisposition to the development of DN.
